# Association between folate and glutamine metabolism and prognosis of kidney cancer

**DOI:** 10.3389/fnut.2024.1506967

**Published:** 2025-01-31

**Authors:** Jiaxuan Qin, Chaoyue Ouyang, Xuan Zhuang

**Affiliations:** ^1^Department of Urology Surgery, The First Affiliated Hospital of Xiamen University, Xiamen, Fujian, China; ^2^School of Medicine, Xiamen University, Xiamen, Fujian, China; ^3^Center of Diagnosis and Treatment of Urinary System Diseases, The First Affiliated Hospital of Xiamen University, Xiamen, Fujian, China; ^4^The Key Laboratory of Urinary Tract Tumors and Calculi of Xiamen City, The First Affiliated Hospital of Xiamen University, Xiamen, Fujian, China

**Keywords:** folate, glutamine, prognosis, kidney cancer, kidney renal clear cell carcinoma, bioinformatics

## Abstract

**Background:**

Recent studies have shown that folate metabolism might influence cancer progression by regulating mitochondrial metabolism and glutamine is involved in the development and progression of several malignancies. This study aimed to explore the association between folate and glutamine metabolism and prognosis of kidney cancer.

**Methods:**

We performed expression analysis, survival analysis, genetic alteration analysis, and tumor immune infiltrate analysis of related genes using platforms such as UALCAN, GEPIA, GEPIA2, cBioPortal, and TIMER. Serum folate, vitamin B12, and methylmalonic acid levels and clinical information of participants in the United States diagnosed with kidney cancer was obtained from the National Health and Nutrition Examination Survey (NHANES) and analyzed using software R.

**Results:**

We observed that RNA expression levels of certain folate and glutamine metabolism-related genes, particularly MTHFD2 and SLC1A5, were associated with the prognosis of kidney renal clear cell carcinoma (KIRC). Expression differences in these genes were notable between high-stage and low-stage and N1 vs. N0 lymph node metastasis status in KIRC. There was a positive association between glutamine metabolism-related genes and folate metabolism-related genes in KIRC. SLC1A5 was positively correlated with MTHFD2 in KIRC. Folate and glutamine metabolism might play a synchronous role in KIRC prognosis. Strong correlations between MTHFD2 and SLC1A5 expression with KIRC immune infiltrates were found. Higher levels of serum folate may be related to improved cancer-specific survival (CSS) in kidney cancer patients in the U.S.

**Conclusion:**

Folate and glutamine metabolism-related genes, especially SLC1A5 and MTHFD2, were associated with the prognosis, tumor stage, and lymph node metastasis status in KIRC. Higher KIRC SLC1A5 or MTHFD2 expression levels were associated with higher tumor stages, increased lymph node metastasis possibilities, poorer OS, and poorer RFS. Elevated levels of serum folate may be associated with improved CSS in kidney cancer patients in the United States.

## 1 Introduction

Metabolic abnormalities are a notable characteristic of tumors. Metabolic properties may evolve during the progression of kidney cancer. Notably, mitochondrial complex I promotes kidney cancer metastasis (1). Additionally, recent studies have shown that folate metabolism might influence cancer progression by regulating mitochondrial metabolism (2).

Plasma folate levels may alter the associations between 5-methyl-2-deoxycytidine, a global DNA methylation marker, blood cadmium concentrations, and kidney cancer (3). Glutamine is closely associated with the mitochondrial tricarboxylic acid (TCA) cycle and involved in the development and progression of several malignancies (4). Many cancer cells depend on glutamine for their growth and proliferation, a phenomenon referred to as “glutamine addiction” (5). Moreover, glutamine metabolism plays a significant role in the prognosis of advanced kidney cancer and in the sunitinib resistance to the drug sunitinib (6).

In one-carbon metabolism, one-carbon units must bind to tetrahydrofolic acid (FH4), which is the active form of folate, in order to be transported. Vitamin B12 serves as a coenzyme in the conversion of folate to FH4 (7, 8).

Vitamin B12 deficiency can lead to increased levels of methylmalonic acid, which serves as a biomarker for assessing vitamin B12 status (9). Methylenetetrahydrofolate dehydrogenase 1 (MTHFD1), MTHFD2, methylenetetrahydrofolate reductase (MTHFR), and 5-methyltetrahydrofolate-homocysteine methyltransferase reductase (MTRR) are important regulatory enzymes in folate metabolism (10–12). The solute carrier family 1 member 5 (SLC1A5) is responsible for transporting glutamine into the cell cytosol, while glutaminase 1 (GLS1) deaminates glutamine in the mitochondria. Subsequently, glutamine is catalyzed by glutamate dehydrogenase 1 (GLUD1) to produce α-ketoglutarate, which then participates in the TCA cycle (13).

In this study, we attempted to explore the association between folate and glutamine metabolism and prognosis of kidney cancer. The correlation between expression levels of genes encoding key regulatory proteins in folate and glutamine metabolism and kidney renal clear cell carcinoma (KIRC) was investigated. Serum folate, vitamin B12, and methylmalonic acid levels and kidney cancer prognosis were evaluated based on the data from the National Health and Nutrition Examination Survey (NHANES). The NHANES offers high-quality and comprehensive data on health and nutritional status of the population, as well as related survival information. This provides reliable evidence to support our study.

## 2 Materials and methods

### 2.1 Data collection in NHANES

Serum folate, vitamin B12, and methylmalonic acid levels and clinical information of participants from the United States diagnosed with kidney cancer, were acquired from the NHANES (https://wwwn.cdc.gov/nchs/nhanes/search/) within the time periods 1999–2004 and 2011–2014. Clinical information included age at screening, age at diagnosis, gender, ethnicity, drinking history, voluntary smoking history, BMI values, follow-up time, overall survival (OS) status, and cancer-specific survival (CSS) status. The NHANES did not provide detailed information on pathological subtypes, TNM staging, tumor stage, or lymph node metastasis status for kidney cancer-diagnosed participants. A total of 55 kidney cancer patients were selected from over 8,000 participants with serum folate, vitamin B12, or methylmalonic acid level data in the NHANES. Related survival data were obtained from the National Death Index (NDI) (https://www.cdc.gov/nchs/data-linkage/mortality-public.htm).

### 2.2 Construction of prognostic folate-related signature based on NHANES data

Vitamin B12 is involved in folate metabolism. Methylmalonic acid can reflect vitamin B12 status. Therefore, we decided to construct a prognostic folate-related signature that comprehensively considers the relationship between kidney cancer prognosis and serum folate, vitamin B12, and methylmalonic acid levels. The coefficient index was achieved using the cph function in the “rms” package of R. Risk score = ∑value_n_ × β_n_. The “value” represents the quartile group value of serum folate, vitamin B12, or methylmalonic acid. The “β” represents the coefficient index value. The “n” represents the serial number. The samples were divided into a low-risk group and a high-risk group based on the median cut-off risk score.

### 2.3 Expression analysis, survival analysis, genetic alteration analysis, and tumor immune infiltrate analysis of related genes in KIRC

Kidney renal clear cell carcinoma (KIRC) is the most common pathological subtype of kidney cancer, and there are differences in gene expression among different pathological subtypes. As a result, we specifically examined KIRC utilizing UALCAN, GEPIA, GEPIA2, cBioPortal, and TIMER, which are all visual online analysis sites. MTHFD1, MTHFD2, MTHFR, and MTRR are important regulatory enzymes in folate metabolism. SLC1A5 plays a crucial role in glutamine transport. GLS1 and GLUD1 are essential for glutamine metabolism. We selected four folate metabolism-related genes and three glutamine metabolism-related genes to analyze the association between folate and glutamine metabolism and KIRC. All these analyses were based on RNA expression data. Detailed clinical information on age, gender, ethnicity, follow-up status, pathological subtypes, TNM staging, tumor stage, and lymph node metastasis status was available in the TCGA KIRC dataset.

Expression analysis and survival analysis of related genes were performed in UALCAN (https://ualcan.path.uab.edu/) and GEPIA (http://gepia.cancer-pku.cn/), based on TCGA KIRC data. Correlation analyses were performed in UALCAN and GEPIA2 (http://gepia2.cancer-pku.cn/), which were also based on TCGA KIRC data. Genetic alteration analysis of related genes was performed in cBioPortal (https://www.cbioportal.org/), which relied on TCGA KIRC data. Tumor immune infiltrate analysis of related genes was performed in TIMER (https://cistrome.shinyapps.io/timer/), based on TCGA KIRC data.

### 2.4 Statistics analysis

R (version 4.3.2) software was used for statistical analyses of NHANES data. Some test methods are annotated in the figures and tables. The correlation was evaluated using the Spearman method. A *P* < 0.05 was defined as statistically significant. Statistical analysis of the online databases is conducted based on the database instructions and the description above. All analyses in GEPIA compared TCGA primary KIRC tissues and TCGA-paired normal tissues.

## 3 Results

### 3.1 Glutamine metabolism-related genes and KIRC prognosis

**Figure 1:**
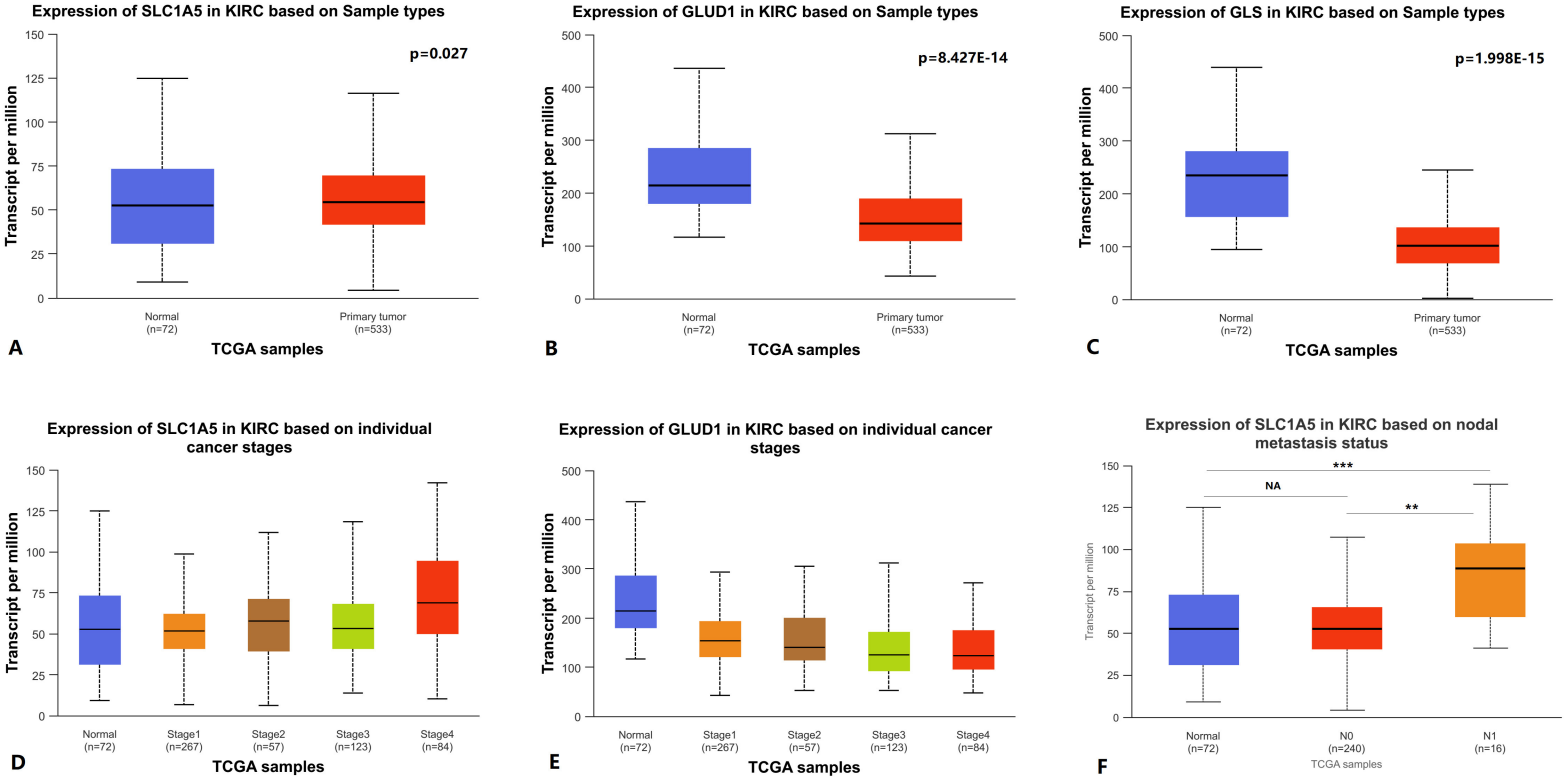
Glutamine metabolism-related genes RNA expression levels in KIRC. **(A–C)** SLC1A5, GLUD1, and GLS1 RNA expression between primary KIRC tissues and paired normal tissues. **(D, E)** SLC1A5 and GLUD1 RNA expression between cancer stages. **(F)** SLC1A5 RNA expression between different lymph node metastasis statuses. KIRC: kidney renal clear cell carcinoma.

SLC1A5 can transport glutamine into the cell cytosol, GLS1 can deaminate glutamine in the mitochondria, and subsequently, glutamine is catalyzed by GLUD1 to produce α-ketoglutarate, which participates in the TCA cycle (13). Using UALCAN to analyze TCGA KIRC RNA expression data, we found that SLC1A5 expression was upregulated in primary KIRC tissues compared to paired normal tissues, while GLUD1 and GLS1 expression was downregulated (Figures 1A–C). The expression level of SLC1A5 was higher in stage 4 vs. stage 1, stage 4 vs. stage 2, and N1 vs. N0 lymph node metastasis status (Figures 1D, F). GLUD1 expression level was lower in stage 4 vs. stage 1 and stage 4 vs. stage 2 (Figure 1E). No statistically significant differences were observed in GLUD1 or GLS1 expression between N1 and N0 lymph node metastasis statuses. Similarly, there were no statistically significant differences in GLS1 expression between stage 4 and stage 1. Detailed *p*-values are shown in Supplementary Table S2.

**Figure 2:**
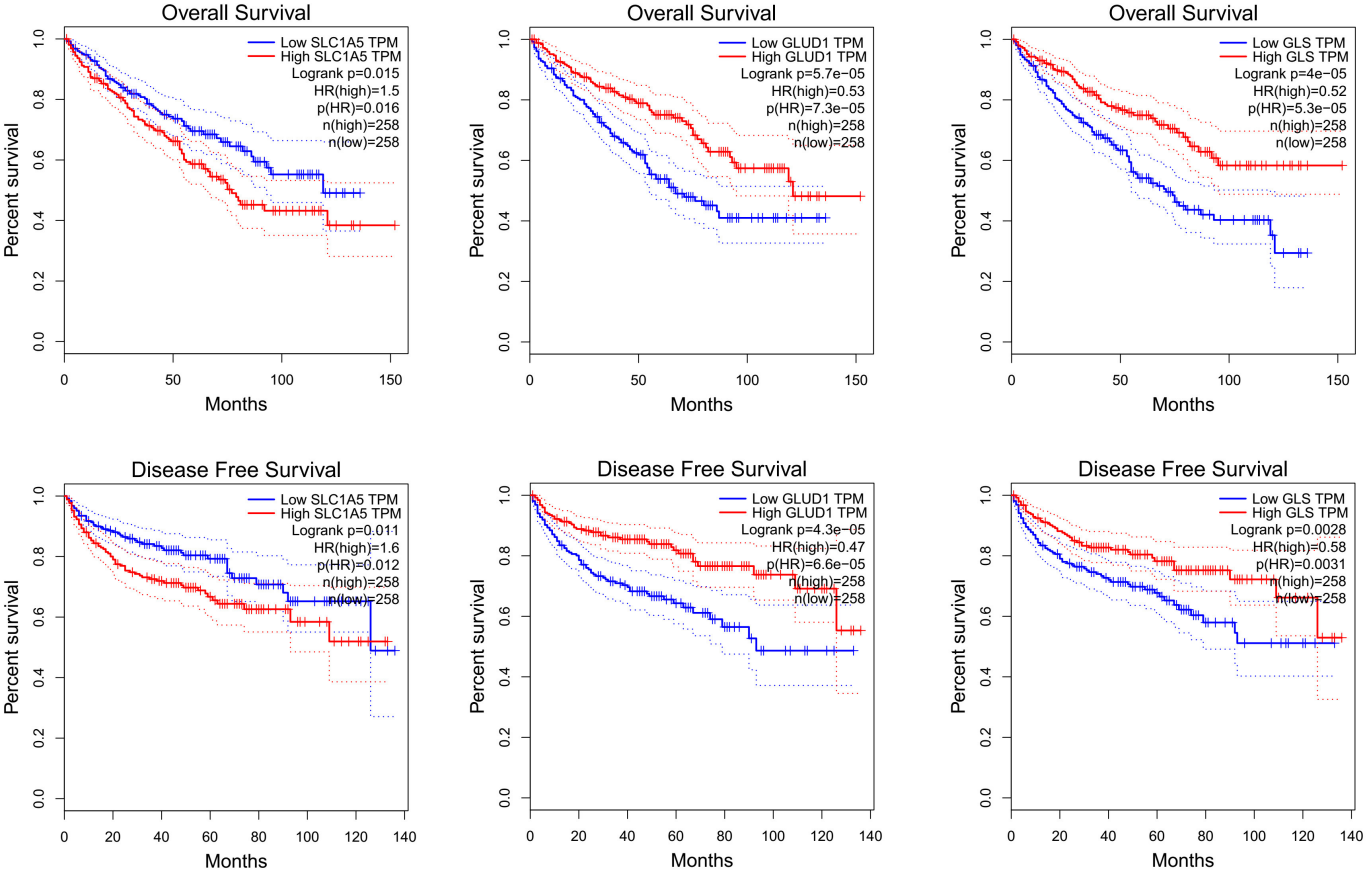
SLC1A5, GLUD1 and GLS1 RNA expression level and KIRC survival: K-M curve of OS and RFS. KIRC: kidney renal clear cell carcinoma.

Using GEPIA to analyze TCGA KIRC RNA expression data, we found that elevated SLC1A5 expression levels were associated with poorer OS and RFS. However, lower GLUD1 and GLS1 expression levels were also associated with poorer OS and RFS (Figure 2). In the analysis, the group cutoff was “median.”

### 3.2 Folate metabolism-related genes and KIRC prognosis

**Figure 3:**
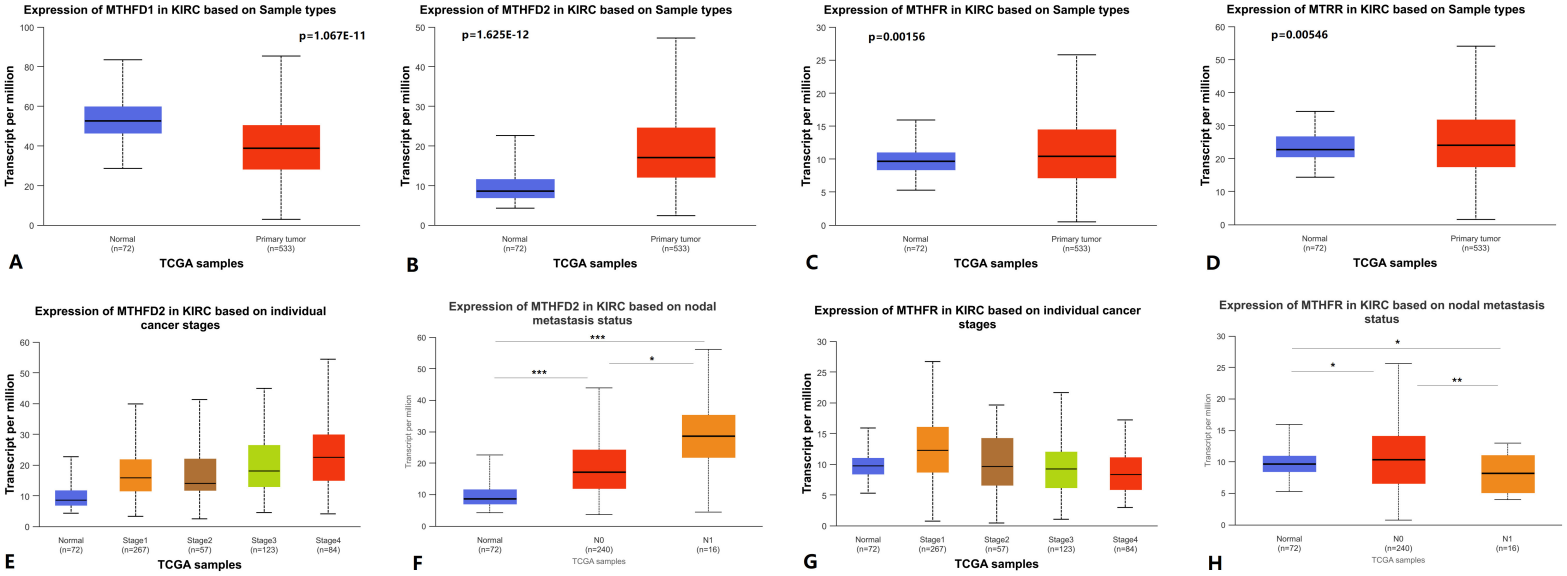
Folate metabolism-related genes RNA expression level in KIRC. **(A–D)** MTHFD1, MTHFD2, MTHFR, and MTRR RNA expression between primary KIRC tissues and paired normal tissues. **(E, G)** MTHFD2 and MTHFR RNA expression between cancer stages. **(F, H)** MTHFD2 and MTHFR RNA expression between different lymph node metastasis status. KIRC: kidney renal clear cell carcinoma.

MTHFD1, MTHFD2, MTHFR, and MTRR are important regulatory enzymes in folate metabolism (10–12). Using UALCAN to analyze TCGA KIRC RNA expression data, we found that the expression of MTHFD2, MTHFR, and MTRR was upregulated in primary KIRC tissues compared to paired normal tissues, while MTHFD1 expression was downregulated (Figures 3A–D). The expression level of MTHFD2 was higher in stage 4 vs. stage 1, stage 4 vs. stage 2, and N1 vs. N0 lymph node metastasis status (Figures 3E, F). The expression level of MTHFR was lower in stage 4 vs. stage 1 and N1 vs. N0 lymph node metastasis status (Figures 3G, H). The expression level of MTHFD1 was lower in N1 vs. N0 lymph node metastasis status (Supplementary Figure S1), but there was no statistically significant difference in stage 4 vs. stage 1. There was no statistically significant difference in MTRR expression in stage 4 vs. stage 1 and N1 vs. N0 lymph node metastasis status. Detailed *p*-values are shown in Supplementary Table S2.

**Figure 4:**
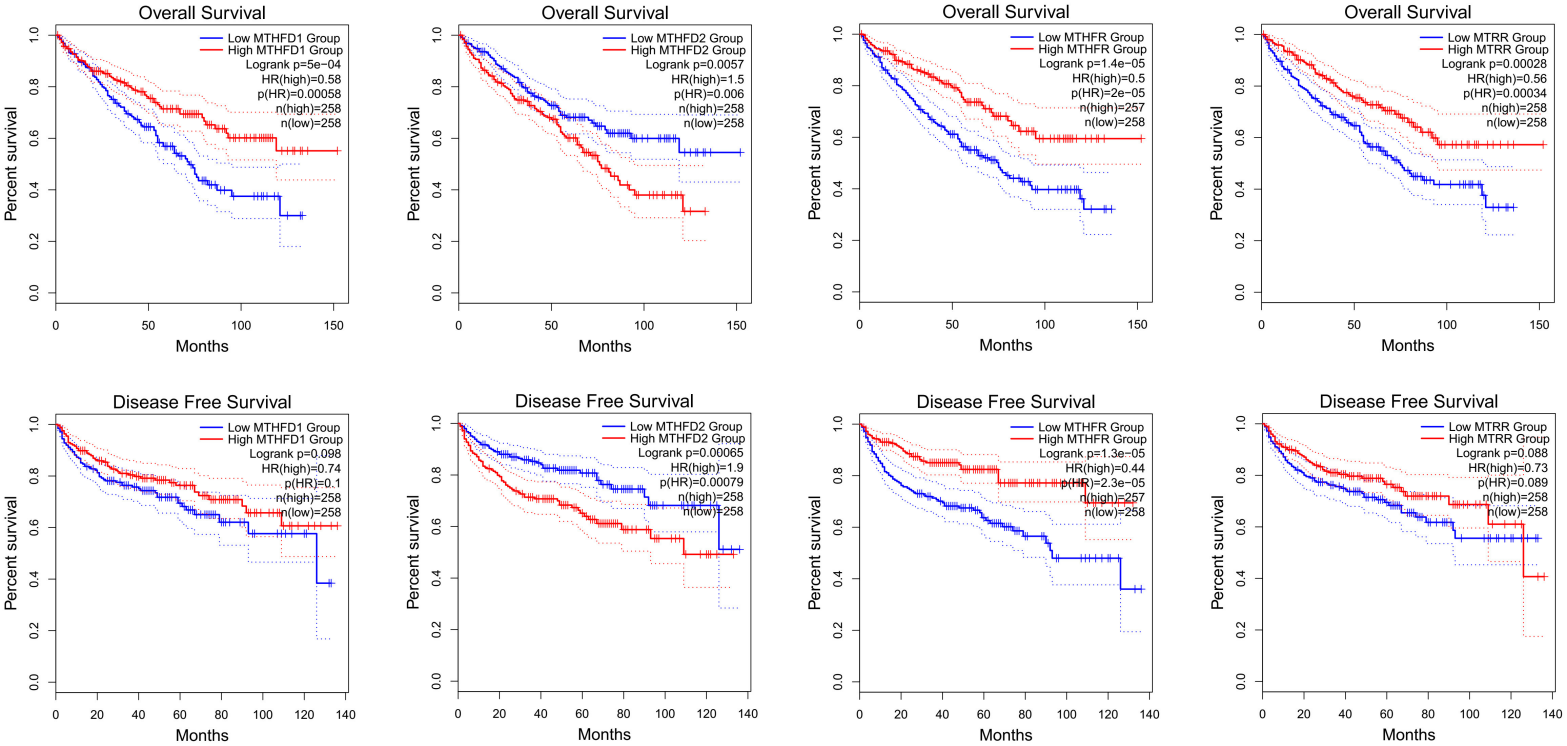
MTHFD1, MTHFD2, MTHFR, and MTRR RNA expression level and KIRC survival, K-M curve of OS and RFS.

Using GEPIA to analyze TCGA KIRC RNA expression data, we found that a higher MTHFD2 expression level was associated with poorer OS and RFS. Lower MTHFR expression level was associated with poorer OS and RFS. Lower MTHFD1 and MTRR expression levels were associated with poorer OS (Figure 4). In the analysis, the group cutoff was “median.”

### 3.3 Association between glutamine metabolism-related genes and folate metabolism-related genes in KIRC

**Figure 5:**
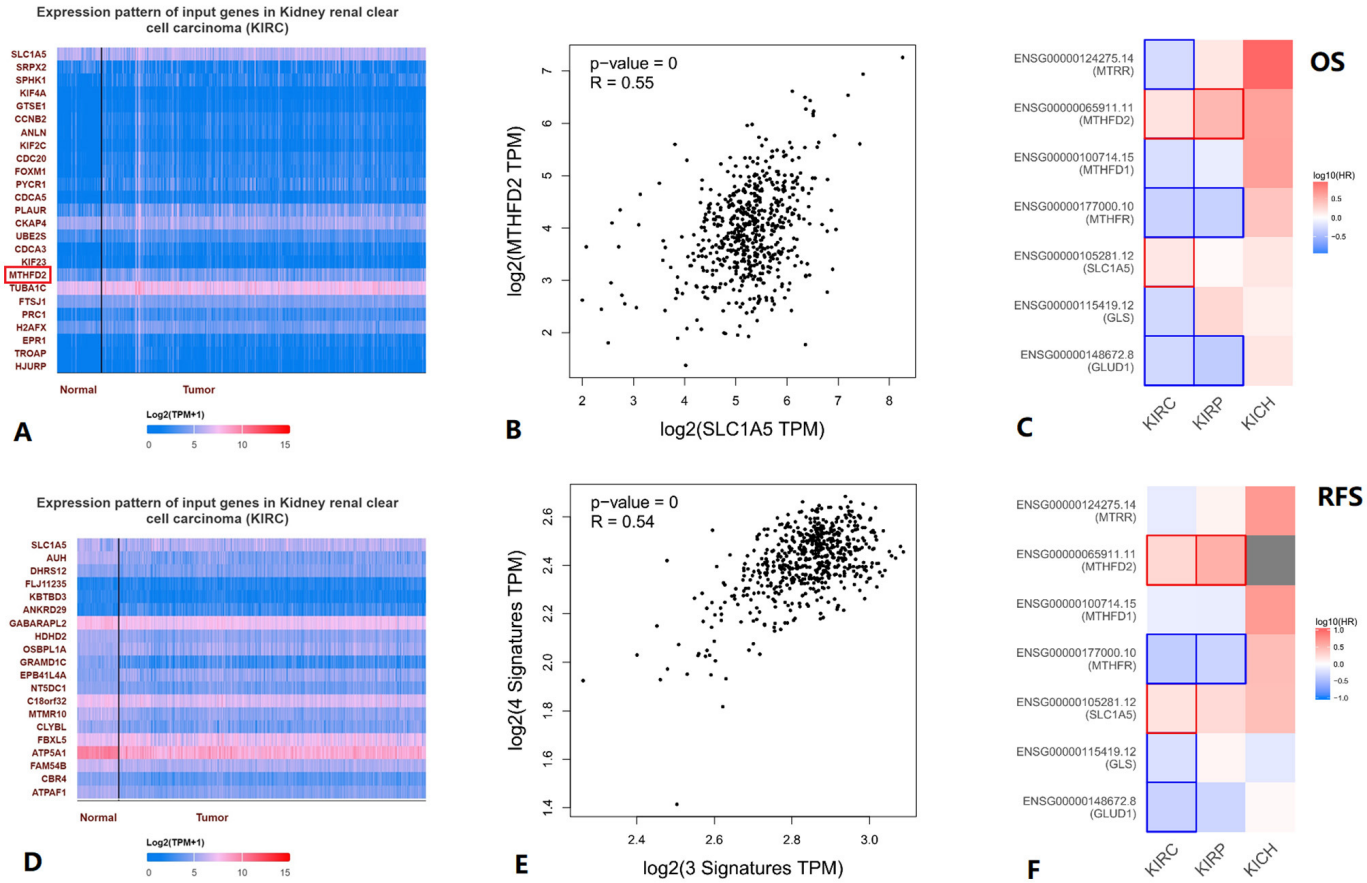
Association between glutamine metabolism-related genes and folate metabolism-related genes in KIRC. **(A)** Top25 genes positively correlated with SLC1A5 in KIRC. **(B)** Correlation between MTHFD2 and SLC1A5 in KIRC. **(C)** Survival map between the seven genes and KIRC OS, statistically significant results are boxed. **(D)** All genes negatively correlated with SLC1A5 in KIRC. **(E)** Correlation between 3 glutamine metabolism-related genes' signature and 4 folate metabolism-related genes' signature in KIRC. **(F)** In the survival map between the seven genes and KIRC RFS, statistically significant results are boxed. KICH, kidney chromophobe; KIRC, kidney renal clear cell carcinoma; KIRP, kidney renal papillary cell carcinoma.

When we used UALCAN to analyze genes correlated with SLC1A5 in KIRC, we found MTHFD2 among the top 25 positively correlated genes (Figures 5A, D). Furthermore, KIRC SLC1A5 was positively correlated with MTHFD2 in the Pearson correlation analysis conducted in GEPIA2 (Figure 5B). A positive correlation between three glutamine metabolism-related gene signatures and four folate metabolism-related gene signatures in KIRC was found through Pearson correlation analysis in GEPIA2 (Figure 5E). Using GEPIA2, we obtained the survival map between the seven genes and kidney cancer OS and RFS, and statistically significant results were boxed in Figures 5C, F.

### 3.4 Genetic alteration and tumor immune infiltrates of MTHFD2 and SLC1A5 in KIRC

**Figure 6:**
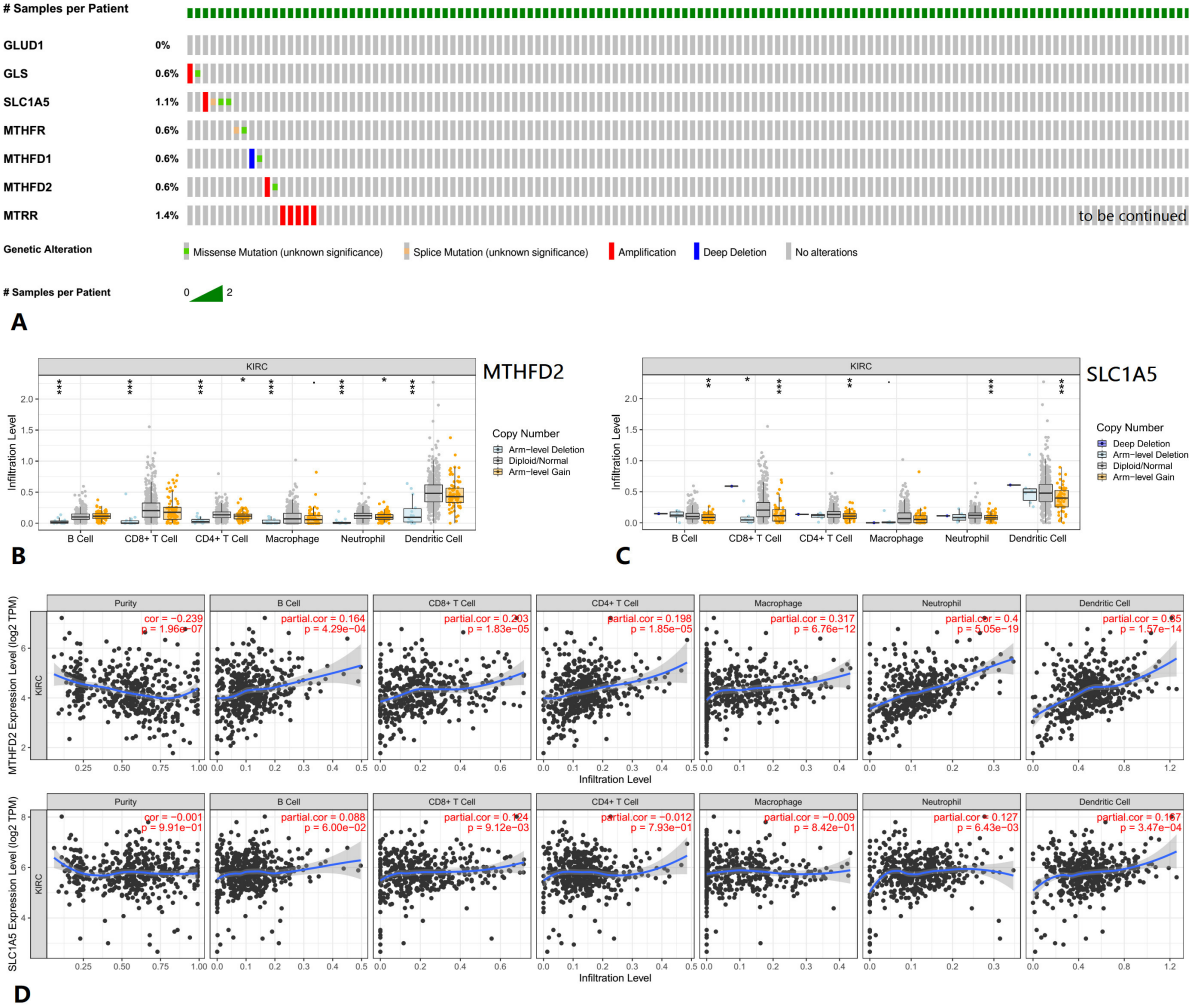
Genetic alteration and tumor immune infiltrates of related genes in KIRC. **(A)** Genetic alteration information of related genes. **(B, C)** Tumor infiltration levels with SCNA for MTHFD2 and SLC1A5. **(D)** Correlations of MTHFD2 and SLC1A5 expression with tumor-infiltrating immune cells. KIRC, kidney renal clear cell carcinoma.

Genetic alteration information from cBioPortal is shown in Figure 6A. Tumor infiltration levels in KIRC with different somatic copy number alterations (SCNA) for MTHFD2 and SLC1A5 are shown in Figures 6B, C. The correlations between MTHFD2 and SLC1A5 expression with selected tumor-infiltrating immune cells in KIRC are shown in Figure 6D.

### 3.5 Serum folate, vitamin B12, and methylmalonic acid levels and kidney cancer prognosis based on the NHANES data

**Figure 7:**
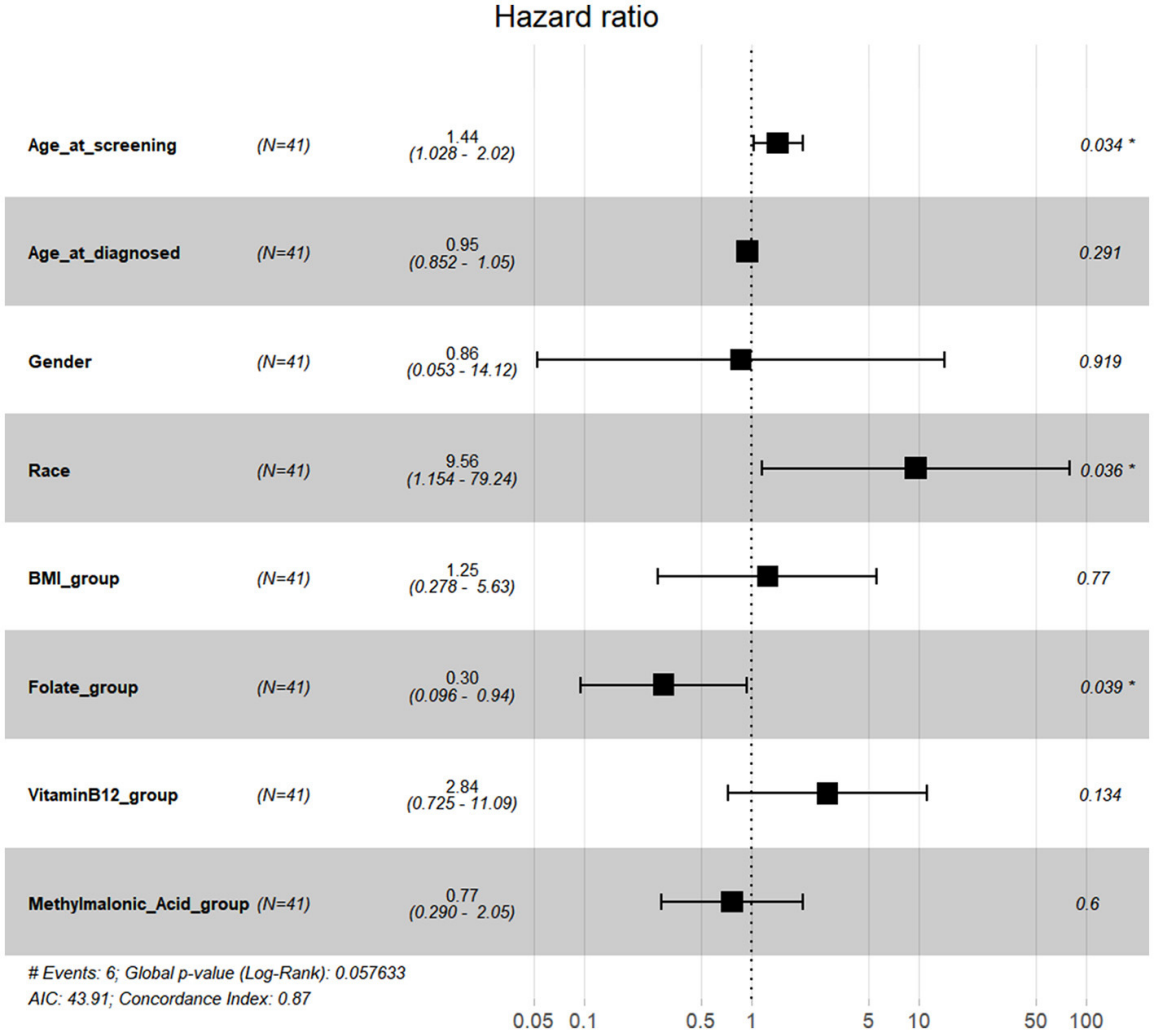
Multivariate Cox regression forest plots with CSS in kidney cancer.

Simultaneously, data on available serum folate, vitamin B12, and methylmalonic acid levels could be found in the NHANES within the time periods 1999–2004 and 2011–2014. We selected 55 US participants with kidney cancer diagnosed within this time period above, but 14 of them lacked sufficient data. Finally, data from 41 participants were used in further analyses. None of the 41 participants had a drinking history or voluntary smoking history. Detailed pathological subtype information was not available. BMI, serum folate, serum vitamin B12, and serum methylmalonic acid values were converted into quartile group values as 1, 2, 3, and 4, respectively. Multivariate Cox regression forest plots were generated using R. No association was found between serum folate, vitamin B12, or methylmalonic acid levels and kidney cancer OS (Supplementary Figure S2). Higher levels of serum folate might be associated with improved CSS, and ethnicity and age at screening might be the confounding factors (Figure 7).

Furthermore, we tried to construct a prognostic folate-related signature, and the coefficient index was calculated using the cph function in the “rms” package in R. Based on OS, we obtained the following formula: Risk score = 0.1211 × Methylmalonic Acid + 0.2529 × Vitamin B12 – 0.4428 × Folate. However, no statistically significant differences in kidney cancer survival were identified between the low- and high-risk groups (Supplementary Table S1). Based on CSS, we obtained the following formula: Risk score = 0.7937 × Vitamin B12 – 1.0694 × Folate – 0.2508 × Methylmalonic Acid. However, no statistically significant result was found, either (Table 1).

**Table 1:** Based on CSS, clinical characteristics between low and high-risk groups in kidney cancer.

**Characteristic**	**High, *N* = 20^a^**	**Low, *N* = 21^a^**	***p*-value^b^**
**Age at screening**			0.22
Mean (SD)	69 (13)	73 (9)	
Median (IQR)	72 (65, 75)	75 (72, 80)	
Range	26, 85	50, 85	
**Age at diagnosed**			0.67
Mean (SD)	60 (15)	63 (11)	
Median (IQR)	61 (50, 70)	63 (57, 69)	
Range	26, 85	48, 85	
**Gender**			0.39
Male	14 (70%)	12 (57%)	
Female	6 (30%)	9 (43%)	
**Race**			0.77
Mexican American	2 (10%)	3 (14%)	
Other Hispanic	0 (0%)	2 (9.5%)	
Non-Hispanic White	12 (60%)	12 (57%)	
Non-Hispanic Black	5 (25%)	3 (14%)	
Other Race	1 (5.0%)	1 (4.8%)	
**BMI group**			>0.99
Underweight	0 (0%)	1 (4.8%)	
Normal weight	2 (10%)	2 (9.5%)	
Overweight	9 (45%)	10 (48%)	
Obesity	9 (45%)	8 (38%)	
OS	10 (50%)	14 (67%)	0.28
CSS	3 (15%)	3 (14%)	>0.99

^a^n (%).

^b^Wilcoxon rank sum test; Pearson's Chi-squared test; Fisher's exact test.

## 4 Discussion

In our study, some folate and glutamine metabolism-related genes' RNA expression levels were associated with KIRC prognosis, especially MTHFD2 and SLC1A5. Meanwhile, we found statistically significant differences in high-stage vs. low-stage and N1 vs. N0 lymph node metastasis status.

Higher expression levels of SLC1A5 or MTHFD2 were associated with poorer OS and relapse-free survival (RFS), advanced tumor stages, and increased likelihood of lymph node metastasis in KIRC patients.

A ‘switch' in mitochondrial status may occur between metastatic and localized kidney cancers. Recent studies have shown that KIRC metastases enhanced TCA cycle labeling compared to primary KIRC, indicating a divergent metabolic programming during metastasis in patients.

Metabolic characteristics may change during the progression of KIRC, with mitochondrial complex I playing a significant role in promoting metastasis (1). Mitochondrial content increased in high-grade KIRC, while the function of mitochondrial respiratory complex II remained low. The progression of KIRC is accompanied by altered mitochondrial respiratory complex II status (14). Mitochondria play a crucial role in tumor cell growth and proliferation by supporting both the ATP synthesis and the production of macromolecular precursors. Altered mitochondrial metabolism contributes to tumor progression (15, 16). Through mitochondrial fission and fusion, cellular ATP demand creates metabolically distinct subpopulations of mitochondria (17). Folate metabolism and glutamine metabolism are closely associated with mitochondrial metabolism. Consequently, these metabolic pathways may exhibit changes accordingly between metastatic kidney cancer and localized kidney cancer.

One-carbon unit metabolism is altered during tumor progression and relies on the combination with the active form of folate (7, 8, 10–12). MTHFD2, a crucial regulatory enzyme in folate metabolism, plays a significant role in one-carbon unit metabolism (18). This one-carbon unit is mainly from amino acids such as serine, tryptophan, histidine, or glycine. In many cancers, the expression of SLC1A5 increases to meet the heightened demand for glutamine caused by rapid cell proliferation (19). Additionally, SLC1A5 is a significant transporter of serine in cancer cells. Serine and glutamine compete for transport through SLC1A5, with SLC1A5-mediated serine uptake being essential for purine nucleotide biosynthesis (20). Both folate metabolism and glutamine metabolism are involved in one-carbon unit metabolism, especially the genes MTHFD2 and SLC1A5. Our study found a positive association between glutamine metabolism-related genes and folate metabolism-related genes in KIRC. SLC1A5 was positively correlated with MTHFD2. Folate metabolism and glutamine metabolism may have interconnected, potentially synchronous roles in influencing KIRC prognosis. Synchronous change of MTHFD2 and SLC1A5 can also be found in mutant p53 breast cancer cell lines (21) and EB virus-infected B cells (22).

MTHFD2 and SLC1A5 expression levels were strongly correlated with KIRC immune infiltrates. Previous studies have shown that tumor microenvironment is associated with the efficacy of immunotherapy (23) and that MTHFD2 might induce cancer immune evasion through PD-L1 upregulation (24). SLC1A5, as an efficient transporter of glutamine, could strengthen the metabolic capabilities and effector functions of tumor-directed CAR-NK and T cells (25).

Based on the NHANES data, higher levels of serum folate might be associated with improved CSS in kidney cancer patients in the United States. We attempted to develop a prognostic folate-related signature. However, no statistically significant correlation was found between the signature and kidney cancer survival. This outcome may have been influenced by the limited sample size.

Additionally, it is important to acknowledge the limitations of this study. The small sample size and limited data necessitate a cautious interpretation of the results.

To summarize, we might estimate KIRC progression and prognosis by monitoring folate metabolism- and glutamine metabolism-related gene expression levels. Higher SLC1A5 or MTHFD2 expression levels were associated with poorer KIRC OS and RFS. Higher SLC1A5 or MTHFD2 expression levels were associated with higher KIRC tumor stage and higher KIRC lymph node metastasis likelihood. Kidney cancer prognosis may potentially be assessed by monitoring serum folate levels, as higher levels appear to be associated with improved CSS. This suggests potential clinical advantages this study might offer for managing KIRC patients.

## 5 Conclusion

Our results suggest that folate metabolism- and glutamine metabolism-related genes, especially SLC1A5 and MTHFD2, are associated with KIRC prognosis, tumor stage, and lymph node metastasis status. Higher KIRC SLC1A5 or MTHFD2 expression levels were associated with higher tumor stages, increased lymph node metastasis possibilities, poorer OS, and poorer RFS. Higher serum folate levels might be associated with better CSS in kidney cancer patients in the US.

## Data Availability

The raw data supporting the conclusions of this article will be made available by the authors, without undue reservation.

## Supplementary material

Supplementary Figure S1 MTHFD1 RNA expression between different lymph node metastasis status in KIRC.

Supplementary Figure S2 Multivariate Cox regression forest plots with OS in kidney cancer.

Supplementary Table S1 Clinical characteristics between low and high risk groups in kidney cancer, based on OS.

Supplementary Table S2 Details of *p*-values in Figures 1, 3.

## References

[1] Bezwada D Perelli L Lesner NP Cai L Brooks B Wu Z. Mitochondrial complex I promotes kidney cancer metastasis. Nature. (2024) 633:923–31. 10.1038/s41586-024-07812-3 39143213 PMC11424252

[2] Lee Y Vousden KH Hennequart M. Cycling back to folate metabolism in cancer. Nat Cancer. 5:701–15. 10.1038/s43018-024-00739-8 38698089 PMC7616045

[3] Huang CY Chen WJ Lee HL Lin YC Huang YL Shiue HS. Possible combined effects of plasma folate levels, global DNA methylation, and blood cadmium concentrations on renal cell carcinoma. Nutrients. (2023) 15:937. 10.3390/nu15040937 36839294 PMC9959822

[4] Erb HHH Polishchuk N Stasyk O Kahya U Weigel MM Dubrovska A. Glutamine metabolism and prostate cancer. Cancers. (2024) 16:2871. 10.3390/cancers16162871 39199642 PMC11352381

[5] Scalise M Pochini L Galluccio M Console L Indiveri C. Glutamine transport and mitochondrial metabolism in cancer cell growth. Front Oncol. (2017) 7:306. 10.3389/fonc.2017.00306 29376023 PMC5770653

[6] Morozumi K Kawasaki Y Sato T Maekawa M Takasaki S Shimada S. Elucidation and regulation of tyrosine kinase inhibitor resistance in renal cell carcinoma cells from the perspective of glutamine metabolism. Metabolites. (2024) 14:170. 10.3390/metabo14030170 38535330 PMC10971907

[7] Pentieva K Caffrey A Duffy B Ward M Clements M Kerr M. B-vitamins and one-carbon metabolism during pregnancy: health impacts and challenges. Proc Nutr Soc. (2024) 2024:1–15. 10.1017/S0029665124004865 39311046

[8] Sobral AF Cunha A Silva V Gil-Martins E Silva R Barbosa DJ. Unveiling the therapeutic potential of folate-dependent one-carbon metabolism in cancer and neurodegeneration. Int J Mol Sci. (2024) 25:9339. 10.3390/ijms25179339 39273288 PMC11395277

[9] Tejero J Lazure F Gomes AP. Methylmalonic acid in aging and disease. Trends Endocrinol Metab. (2024) 35:188–200. 10.1016/j.tem.2023.11.001 38030482 PMC10939937

[10] Pietruszyńska-Reszetarska A Pietruszyński R Irzmański R. The significance of genetically determined methylation and folate metabolism disorders in the pathogenesis of coronary artery disease: a target for new therapies? Int J Mol Sci. (2024) 25:6924. 10.3390/ijms25136924 39000032 PMC11241586

[11] Karas KuŽelički N Doljak B. Congenital heart disease and genetic changes in folate/methionine cycles. Genes (Basel). (2024) 15:872. 10.3390/genes15070872 39062651 PMC11276067

[12] Liu H Ou J Chen Y Chen Q Luo M Wang T. Association of maternal folate intake and offspring MTHFD1 and MTHFD2 genes with congenital heart disease. Nutrients. (2023) 15:3502. 10.3390/nu15163502 37630697 PMC10458540

[13] Yoo HC Yu YC Sung Y Han JM. Glutamine reliance in cell metabolism. Exp Mol Med. (2020) 52:1496–516. 10.1038/s12276-020-00504-8 32943735 PMC8080614

[14] Miklovicova S Volpini L Sanovec O Monaco F Vanova KH Novak J. Mitochondrial respiratory complex II is altered in renal carcinoma. Biochim Biophys Acta Mol Basis Dis. (2025) 1871:167556. 10.1016/j.bbadis.2024.167556 39486656

[15] Valcarcel-Jimenez L Gaude E Torrano V Frezza C Carracedo A. Mitochondrial metabolism: Yin and Yang for tumor progression. Trends Endocrinol Metab. (2017) 28:748–57. 10.1016/j.tem.2017.06.004 28938972 PMC6047739

[16] Chakraborty S Balan M Sabarwal A Choueiri TK Pal S. Metabolic reprogramming in renal cancer: events of a metabolic disease. Biochim Biophys Acta Rev Cancer. (2021) 1876:188559. 10.1016/j.bbcan.2021.188559 33965513 PMC8349779

[17] Ryu KW Fung TS Baker DC Saoi M Park J Febres-Aldana CA. Cellular ATP demand creates metabolically distinct subpopulations of mitochondria. Nature. (2024) 635:746–54. 10.1038/s41586-024-08146-w 39506109 PMC11869630

[18] Ducker GS Chen L Morscher RJ Ghergurovich JM Esposito M Teng X. Reversal of cytosolic one-carbon flux compensates for loss of the mitochondrial folate pathway. Cell Metab. (2016) 23:1140–53. 10.1016/j.cmet.2016.04.016 27211901 PMC4909566

[19] Scalise M Pochini L Console L Losso MA Indiveri C. The human SLC1A5 (ASCT2) amino acid transporter: from function to structure and role in cell biology. Front Cell Dev Biol. (2018) 6:96. 10.3389/fcell.2018.00096 30234109 PMC6131531

[20] Conger KO Chidley C Ozgurses ME Zhao H Kim Y Semina SE. ASCT2 is a major contributor to serine uptake in cancer cells. Cell Rep. (2024) 43:114552. 10.1016/j.celrep.2024.114552 39068660 PMC11406281

[21] Tombari C Zannini A Bertolio R Pedretti S Audano M Triboli L. Mutant p53 sustains serine-glycine synthesis and essential amino acids intake promoting breast cancer growth. Nat Commun. (2023) 14:6777. 10.1038/s41467-023-42458-1 37880212 PMC10600207

[22] Wang LW Shen H Nobre L Ersing I Paulo JA Trudeau S. Epstein-Barr-virus-induced one-carbon metabolism drives B cell transformation. Cell Metab. (2019) 30:539–55.e11. 10.1016/j.cmet.2019.06.003 31257153 PMC6720460

[23] Kim J Seki E. Inflammation and immunity in liver neoplasms: implications for future therapeutic strategies. Mol Cancer Ther. (2024). 10.1158/1535-7163.MCT-23-0726. [Epub ahead of print]. 39365846 PMC11794036

[24] Shang M Yang H Yang R Chen T Fu Y Li Y. The folate cycle enzyme MTHFD2 induces cancer immune evasion through PD-L1 up-regulation. Nat Commun. (2021) 12:1940. 10.1038/s41467-021-22173-5 33782411 PMC8007798

[25] Nachef M Ali AK Almutairi SM Lee SH. Targeting SLC1A5 and SLC3A2/SLC7A5as a potential strategy to strengthen anti-tumor immunity in the tumor microenvironment. Front Immunol. (2021) 12:624324. 10.3389/fimmu.2021.624324 33953707 PMC8089370

