# Supplementary figures and images for: Association between folate and glutamine metabolism and prognosis of kidney cancer
Source: Front Nutr. 2025 Jan 31;11:1506967. doi: 10.3389/fnut.2024.1506967 (PMC11825324; doi:10.3389/fnut.2024.1506967)

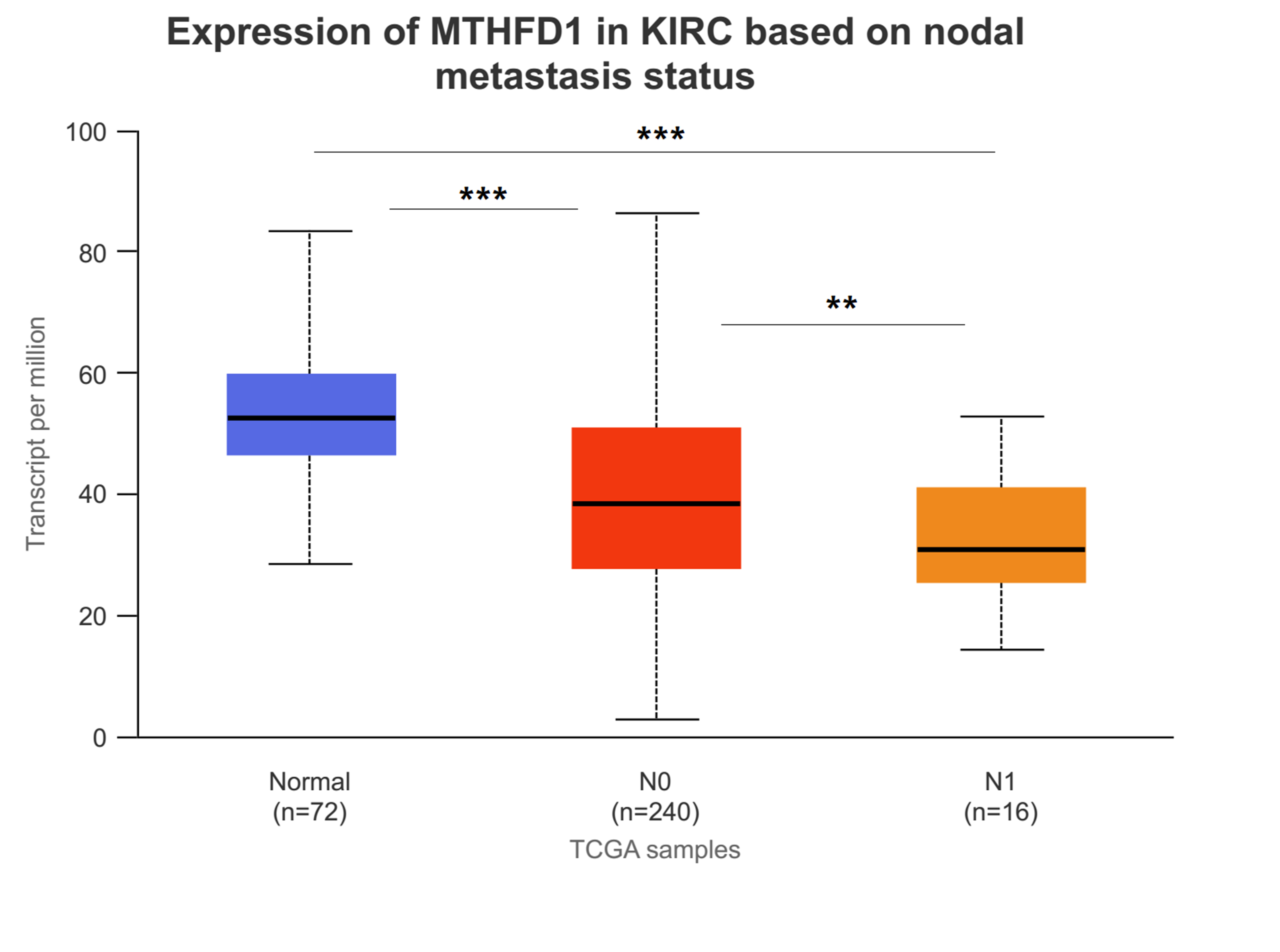

Supplement: Supplementary Figure S1 — MTHFD1 RNA expression between different lymph node metastasis status in KIRC. [file Image_1.tif]

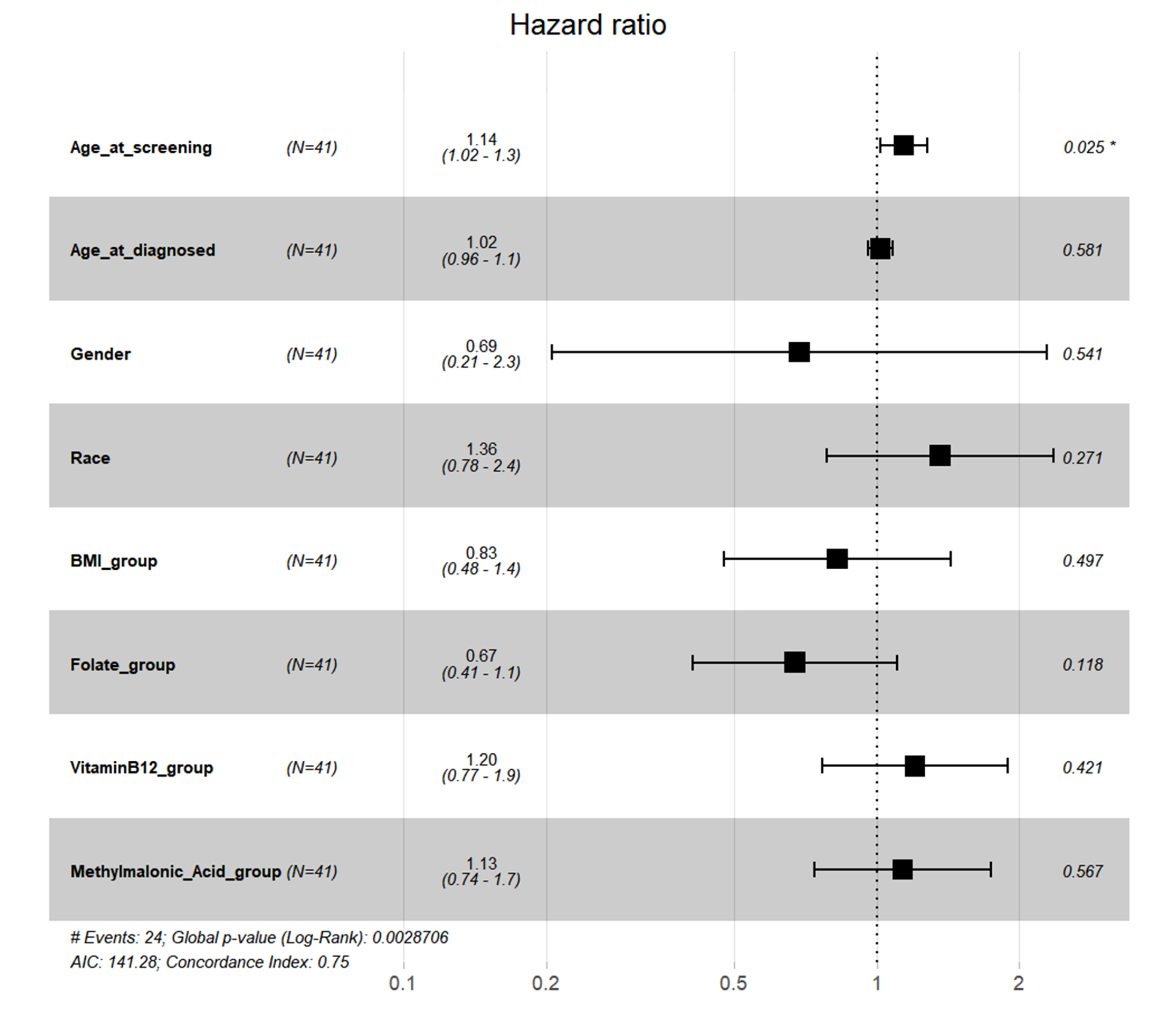

Supplement: Supplementary Figure S2 — Multivariate Cox regression forest plots with OS in kidney cancer. [file Image_2.tiff]
