# Supplementary material for: Association between folate and glutamine metabolism and prognosis of kidney cancer
Source: Front Nutr. 2025 Jan 31;11:1506967. doi: 10.3389/fnut.2024.1506967 (PMC11825324; doi:10.3389/fnut.2024.1506967)
Supplement: Supplementary Table S1 — Clinical characteristics between low and high risk groups in kidney cancer, based on OS. [file Table_1.docx]

**Table S1.** Clinical characteristics between low and high risk groups in kidney cancer, based on OS.

| **Characteristic** | **High** **Risk**, N = 21*^1^* | **Low Risk**, N = 20*^1^* | **p-value***^2^* |
| --- | --- | --- | --- |
| Age at screening |  |  | 0.17 |
| Mean (SD) | 68 (14) | 73 (8) |  |
| Median (IQR) | 72 (65, 76) | 75 (72, 79) |  |
| Range | 26, 85 | 50, 84 |  |
| Age at diagnosed |  |  | >0.99 |
| Mean (SD) | 61 (16) | 62 (10) |  |
| Median (IQR) | 63 (49, 71) | 62 (57, 68) |  |
| Range | 26, 85 | 48, 80 |  |
| Gender |  |  | 0.39 |
| Male | 12 (57%) | 14 (70%) |  |
| Female | 9 (43%) | 6 (30%) |  |
| Race |  |  | 0.6 |
| Mexican American | 3 (14%) | 2 (10%) |  |
| Other Hispanic | 1 (4.8%) | 1 (5.0%) |  |
| Non-Hispanic White | 10 (48%) | 14 (70%) |  |
| Non-Hispanic Black | 5 (24%) | 3 (15%) |  |
| Other Race | 2 (9.5%) | 0 (0%) |  |
| BMI |  |  | >0.99 |
| Underweight | 1 (4.8%) | 0 (0%) |  |
| Normal weight | 2 (9.5%) | 2 (10%) |  |
| Overweight | 10 (48%) | 9 (45%) |  |
| Obesity | 8 (38%) | 9 (45%) |  |
| OS | 12 (57%) | 12 (60%) | 0.85 |
| CSS | 3 (14%) | 3 (15%) | >0.99 |
| *^1^* n (%) | | | |
| *^2^* Wilcoxon rank sum test; Pearson’s Chi-squared test; Fisher’s exact test | | | |
