# Supplementary material for: Association between folate and glutamine metabolism and prognosis of kidney cancer
Source: Front Nutr. 2025 Jan 31;11:1506967. doi: 10.3389/fnut.2024.1506967 (PMC11825324; doi:10.3389/fnut.2024.1506967)
Supplement: Supplementary Table S2 — Details of p-values in Figures 1, 3. [file Table_2.docx]

**Table S2.** Details of p values in Figure 1 and Figure 3.

1. KIRC SLC1A5 expression in STAGEs

| **Comparison** | **Statistical significance** |
| --- | --- |
| Normal-vs-Stage1 | 5.784200E-01 |
| Normal-vs-Stage2 | 5.307600E-01 |
| **Normal-vs-Stage3** | **3.537700E-02*** |
| **Normal-vs-Stage4** | **5.62079999999465E-05***** |
| Stage1-vs-Stage2 | 7.875400E-01 |
| **Stage1-vs-Stage3** | **3.491800E-02*** |
| **Stage1-vs-Stage4** | **4.07410000000663E-05***** |
| Stage2-vs-Stage3 | 1.489180E-01 |
| **Stage2-vs-Stage4** | **3.375200E-04***** |
| **Stage3-vs-Stage4** | **9.378500E-03**** |

2. KIRC SLC1A5 expression in lymph node metastasis

| **pathologic_N descriptions** | | | |
| --- | --- | --- | --- |
| **N0** | No regional lymph node metastasis | **N1** | Metastases in 1 to 3 axillary lymph nodes |
| **N2** | Metastases in 4 to 9 axillary lymph nodes | **N3** | Metastases in 10 or more axillary lymph nodes |

| \| **Hide statistics** \| \| \| --- \| --- \| \| **Comparison** \| **Statistical significance** \| \| Normal-vs-N0 \| 1.144800E-01 \| \| **Normal-vs-N1** \| **7.64550000000863E-05***** \| \| **N0-vs-N1** \| **9.141600E-03**** \| |
| --- | --- | --- | --- | --- | --- | --- | --- | --- | --- | --- |

3. KIRC GLUD1 expression in STAGEs

| **Comparison** | **Statistical significance** |
| --- | --- |
| **Normal-vs-Stage1** | **2.39410002933482E-09***** |
| **Normal-vs-Stage2** | **7.50949999961392E-07***** |
| **Normal-vs-Stage3** | **6.81454892514921E-13***** |
| **Normal-vs-Stage4** | **1.64079860809352E-12***** |
| Stage1-vs-Stage2 | 7.374200E-01 |
| **Stage1-vs-Stage3** | **6.497800E-03**** |
| **Stage1-vs-Stage4** | **1.383700E-03**** |
| Stage2-vs-Stage3 | 1.152030E-01 |
| **Stage2-vs-Stage4** | **2.766400E-02*** |
| Stage3-vs-Stage4 | 4.868600E-01 |

4. KIRC MTHFD2 expression in STAGEs

| **Comparison** | **Statistical significance** |
| --- | --- |
| **Normal-vs-Stage1** | **4.12440082087073E-11***** |
| **Normal-vs-Stage2** | **8.814800E-04***** |
| **Normal-vs-Stage3** | **2.12920003850314E-09***** |
| **Normal-vs-Stage4** | **8.78229999834446E-09***** |
| Stage1-vs-Stage2 | 9.353200E-01 |
| **Stage1-vs-Stage3** | **4.900400E-02*** |
| **Stage1-vs-Stage4** | **8.555000E-04***** |
| Stage2-vs-Stage3 | 1.817380E-01 |
| **Stage2-vs-Stage4** | **4.235200E-03**** |
| Stage3-vs-Stage4 | 5.201900E-02 |

5. KIRC MTHFD2 expression in lymph node metastasis

| **pathologic_N descriptions** | | | |
| --- | --- | --- | --- |
| **N0** | No regional lymph node metastasis | **N1** | Metastases in 1 to 3 axillary lymph nodes |
| **N2** | Metastases in 4 to 9 axillary lymph nodes | **N3** | Metastases in 10 or more axillary lymph nodes |

| \| **Hide statistics** \| \| \| --- \| --- \| \| **Comparison** \| **Statistical significance** \| \| **Normal-vs-N0** \| **5.22393239776875E-12***** \| \| **Normal-vs-N1** \| **3.610000E-04***** \| \| **N0-vs-N1** \| **1.123960E-02*** \| |
| --- | --- | --- | --- | --- | --- | --- | --- | --- | --- | --- |

6. KIRC MTHFR expression in STAGEs

| **Comparison** | **Statistical significance** |
| --- | --- |
| **Normal-vs-Stage1** | **4.30330000522616E-08***** |
| Normal-vs-Stage2 | 4.110800E-01 |
| Normal-vs-Stage3 | 7.642000E-01 |
| Normal-vs-Stage4 | 1.071800E-01 |
| **Stage1-vs-Stage2** | **2.394100E-02*** |
| **Stage1-vs-Stage3** | **8.46620000000131E-05***** |
| **Stage1-vs-Stage4** | **6.22870000022147E-09***** |
| Stage2-vs-Stage3 | 5.568600E-01 |
| Stage2-vs-Stage4 | 7.867800E-02 |
| Stage3-vs-Stage4 | 1.196570E-01 |

7. KIRC MTHFR expression in lymph node metastasis

| **pathologic_N descriptions** | | | |
| --- | --- | --- | --- |
| **N0** | No regional lymph node metastasis | **N1** | Metastases in 1 to 3 axillary lymph nodes |
| **N2** | Metastases in 4 to 9 axillary lymph nodes | **N3** | Metastases in 10 or more axillary lymph nodes |

| \| **Hide statistics** \| \| \| --- \| --- \| \| **Comparison** \| **Statistical significance** \| \| **Normal-vs-N0** \| **3.868400E-02*** \| \| **Normal-vs-N1** \| **1.700440E-02*** \| \| **N0-vs-N1** \| **3.117000E-03**** \| |
| --- | --- | --- | --- | --- | --- | --- | --- | --- | --- | --- |
